# *S. aureus* blocks efferocytosis of neutrophils by macrophages through the activity of its virulence factor alpha toxin

**DOI:** 10.1038/srep35466

**Published:** 2016-10-14

**Authors:** Taylor S. Cohen, Omari Jones-Nelson, Meghan Hotz, Lily Cheng, Lloyd S. Miller, JoAnn Suzich, C. Kendall Stover, Bret R. Sellman

**Affiliations:** 1 Department of Infectious Disease, Medimmune, LLC One MedImmune Way, Gaithersburg, MD 20878, USA; 2 Department of Translational Science, Medimmune, LLC One MedImmune Way, Gaithersburg, MD 20878, USA; 3 Department of Dermatology, John Hopkins University School of Medicine, Baltimore MD 21231, USA.

## Abstract

Bacterial pneumonia, such as those caused by *Staphylococcus aureus*, is associated with an influx of inflammatory neutrophils into the lung tissue and airways. Regulation and clearance of recruited neutrophils is essential for preventing tissue damage by “friendly fire”, a responsibility of macrophages in a process called efferocytosis. We hypothesized that *S. aureus* impairs efferocytosis by alveolar macrophages (AMs) through the activity of the secreted virulence factor alpha toxin (AT), which has been implicated in altering the antimicrobial function of AMs. Infection of mice lacking AMs resulted in significantly increased numbers of neutrophils in the lung, while clearance of neutrophils delivered intranasally into uninfected mice was reduced in AM depleted animals. *In vitro*, sublytic levels of AT impaired uptake of apoptotic neutrophils by purified AMs. *In vivo*, the presence of AT reduced uptake of neutrophils by AMs. Differential uptake of neutrophils was not due to changes in either the CD47/CD172 axis or CD36 levels. AT significantly reduced lung expression of CCN1 and altered AM surface localization of DD1α, two proteins known to influence efferocytosis. We conclude that AT may contribute to tissue damage during *S. aureus* pneumonia by inhibiting the ability of AM to clear neutrophils at the site of infection.

*Staphylococcus aureus* is an opportunistic pathogen, and has been implicated in a variety of diseases including skin and soft tissue infections and more life threatening diseases such as endocarditis, sepsis, and necrotizing pneumonia. The high frequency of community and hospital associated *S. aureus* infections is a major health concern, and is further exacerbated by the emergence of drug resistant strains.

The success of this pathogen is due in part to its production of a diverse array of virulence factors including multiple pore-forming toxins. Of these, alpha toxin (AT) is a significant determinant of bacteria virulence, particularly in pneumonia, and is expressed by the vast majority of *S. aureus* isolates^1^. AT binds to the metallaprotease ADAM10 on the surface of host cells and oligomerizes into a heptameric transmembrane pore in the mammalian cell membrane^2^. At sublytic concentrations, AT pore formation results in changes in intracellular ion concentration and inflammatory signaling activation (inflammasome), whereas higher AT concentrations lead to cell lysis and possibly hyper-inflammation of the lung^3^,^4^. Clinically, AT expression correlates with severity of infection, and monoclonal antibodies (mAbs) targeting AT increase survival and bacterial clearance in pre-clinical murine pneumonia models and are currently in clinical trials for the prevention of *S. aureus* pneumonia^5^,^6^.

Lung infection by *S. aureus* initiates a rapid innate immune response, including recruitment of phagocytic cells such as neutrophils to the area of infection^7^. Neutrophils are considered as essential components of the innate response to bacterial pathogens, defending against bacterial infection through phagocytic killing, production of neutrophil extracellular traps (NETs), and secretion of inflammatory cytokines which recruit additional phagocytes^8^. While these bactericidal processes are required for optimal bacterial clearance, excessive recruitment and activation of these cells can lead to tissue damage^9^,^10^,^11^,^12^. Furthermore, *S. aureus* is capable of surviving within neutrophils, thereby concealing itself and preventing clearance by other phagocytes^13^.

In addition to clearing microbial pathogens, macrophages and recruited monocytes also clear dying neutrophils through a process called efferocytosis that is mediated by a wide variety of host receptors, recently reviewed by Arandjelovic and Ravichandran^14^. Since, *S. aureus* has been shown to survive within neutrophils; removal of infected neutrophils by other phagocytes is likely essential for resolving the infection^13^. One mechanism by which *S. aureus* has been shown to interfere with this clearance process is by inducing upregulation of the “don’t eat me” signal CD47 on infected neutrophils, which binds macrophage expressed CD172 (Signal regulatory protein α, SIRPα), preventing efferocytosis^15^. However, the bacterial mechanisms that regulate macrophage efferocytosis of neutrophils from *S. aureus* infected lungs are not entirely clear.

Given AT’s effects on macrophages we investigated whether an AT mediated mechanism also contributes to the inhibition of macrophages to remove dying neutrophils from *S. aureus* infected lungs. Herein, we demonstrate that *S. aureus* AT slows the neutrophil clearance process through direct interaction with the alveolar macrophage. Furthermore, we show that neutralization of AT with the clinical candidate monoclonal antibody MEDI4893* restores normal neutrophil efferocytosis by respiratory macrophages, and identify two potential targets of AT’s anti-efferocytosis activity in the lung. Taken together, we define a previously unrecognized function of AT in inhibiting efferocytosis of neutrophils by AMs, providing a new mechanism to therapeutically target during *S. aureus* pneumonia.

## Materials and Methods

### Reagents

Community acquired methicillian-resistant (CA-MRSA) *S. aureus* SF8300 wild type (WT) and its isogenic mutant ∆*hla* were generously provided by Bihn Diep (University of California). Monoclonal antibodies (mAb) were diluted and prepared fresh daily from refrigerated stocks into sterile phosphate buffer saline (PBS), p.H 7.2 (Invitrogen, Carlsbad CA). The neutralizing alpha toxin monoclonal antibody (mAb) MEDI4893* was previously described^16^. Purification and characterization of alpha toxin (AT) and AT_H35L_ (non-pore forming toxoid) were previously described^17^. Isotype human IgG1 was used as control for studies that included MEDI4893*.

### Pneumonia Model

All animal studies were approved by the MedImmune Institutional Animal Care and Use Committee and were conducted in an Association for Accreditation and Assessment Laboratory Animal Care (AAALAC)-accredited facility in compliance with U.S. regulations governing the housing and use of animals. All experiments were repeated at least 3 times unless noted in figure legend.

Neutrophils were isolated from the bone marrow of female C57BL/6-Tg(CAG-EGFP)1310sb/LeySopJ mice (The Jackson Laboratory) using neutrophil isolation kits (Miltenyl Biotech) according to the manufacturer’s instructions. Purified neutrophils were delivered either intranasally (IN, 5e5 cells/animal) in 50 μl PBS or IV (IV, 5e6 cells/animal) in 100 μl PBS. SF8300 stocks were thawed and diluted to the appropriate inoculum in sterile PBS, p.H 7.2 (Invitrogen). Colony forming units (CFU) of challenge inoculum were confirmed by serial dilutions onto trypticase soy agar (TSA) plates (BBL, Becton, Dickinson laboratories) and incubated overnight at 37 °C. All bacterial suspensions and toxins were intranasally administered in 50 μl of PBS.

Specific-pathogen-free 7- to 8-week-old female C57BL/6J mice (The Jackson Laboratory) were anesthetized and maintained in 3% isoflurane (Butler Schein™ Animal Health) with oxygen at 3 L/min and infected IN with *S.aureus* bacteria, AT or recombinant AT_H35L_. Twenty-four h post infection, animals were euthanized by CO_2_, the lungs were perfused with 5 ml of PBS and bronchial alveolar lavage (BAL) and lung tissue was collected for analysis of immune cell populations. Histopathology was previously described^16^. In select experiments alveolar macrophages were depleted 24 h prior to infection via intra-nasal inoculation with 50 μL clodronate liposomes as previously described^17^; control mice received PBS filled liposomes (clodronateliposomes.com).

### Measurement of Myeloperoxidase and Neutrophil Elastase Activity

Myeloperoxidase activity was measured 24 h post infection. Mice were IV injected with either xenolight rediject inflammatory probe (Perkin Elmer, Inc) 5 min before euthanasia and lung excision or neutrophil elastase 680 FAST (Perkin Elmer, Inc) 4 h before euthanasia and lung excision. Excised lungs were measured for chemiluminscence or epifluorescence using IVIS Spectrum (Perkin Elmer).

### Flow Cytometry

Lungs were homogenized through a 40 μm filter (Corning, Inc.), pelleted (500 × g, 5 min) and washed twice in ice-cold FACs buffer (PBS with 5% fetal bovine serum, and 0.1% sodium azide). Red blood cells were removed with ACK Lysing Buffer (Life Technologies), Fc receptors were blocked with anti-mouse CD16/CD32 (eBioscience) and cells were stained with antibodies against mouse CD68 (PE conjugated, clone FA-11), CD11c (APC-Cy5.5 or FITC conjugated, clone N418), CD11b (BV605 conjugated, clone M1/70), Ly6-G (BV421 or PE-Cy7 conjugated, clone 1A8), CD170(SiglecF) (PerCP-eFlour710 or eFluor660 conjugated, clone 1RNM44N), CD36 (PE conjugated, clone MH36), CD47 (FITC conjugated, clone MIAP301), or CD172(SIRPα) (PerCP-eFlour710 conjugated, clone P84) from eBioscience or BioLegend. Cells were imaged using the LSR II Flow Cytometer (BD Biosciences) and analyzed with FlowJo (FlowJo). A known concentration of counting beads (Bangs Laboratories) was added to each sample to calculate the number of cells.

### *In vitro* efferocytosis assay

Alveolar macrophages (AM), the primary cell type in uninfected BAL (>85%), were recovered from BAL fluid, resuspended in RPMI 1640 with 10% fetal bovine serum (FBS) and penicillin and streptomycin (100 U/ml) and cultured overnight at 37 °C with 5% CO_2_. GFP-neutrophils were isolated as described above and incubated for 4 h in RPMI 1640 with 10% FBS with staurosporine (0.1 μM) to induce apoptosis (~75% Annexin V^+^ Propidium iodide^−^, Supplemental Fig. 1) as previously described^18^. Neutrophils were incubated with AMs (20:1 ratio of neutrophils to AMs) for 2 h, after which the cells were stained with anti-mouse CD68 as described above, and analyzed by FACs. Efferocytosis was monitored as the percentage of CD68^+^ cells (macrophages) which were also GFP^+^. In select experiments AMs were incubated with neutralizing antibody against DD1α (BioLegend) or control IgG for 1 h prior to the addition of neutrophils.

### Confocal Imaging

For imaging, 100 k AM, purified from BAL of uninfected animals, were allowed to adhere to each well of a chamber slide (Corning) overnight. Cells were incubated with GFP-neutrophils (20:1 ratio of neutrophil to macrophage) for 2 h, washed 3x with PBS, fixed in 10% formalin (10 min, VWR International), and DD1α was stained with anti-mouse PD-1H (BioLegend) followed by AF633 anti-armenian hamster IgG (Invitrogen). Slides were sealed with Vectashield with DAPI (Vector Laboratories) and imaged with a Leica TCS SP5 X confocal microscope (Leica Microsystems CMS Gmb).

### Western Blot

Prior to protein separation, lung homogenates were lysed with M-PER mammalian protein extraction reagent (Thermo Scientific) and BAL was concentrated using Amicon Ultra-0.5 mL centrifugal filters with a 3 KDa cut off. Equal amounts of protein (lung) or equal volume (BAL) were were separated on 4–12% bis-Tris NuPAGE gels (Invitrogen), transferred to PVDF membranes (ThermoFisher Scientific). Immunodetection was performed using anti-CCN1 (abcam) and anti-actin (Sigma) antibodies. Proteins were visualized and quantified on with the Odyssey imaging system (Li-COR). Data were normalized to the mean intensity of the protein bands in the c-IgG (*in vivo*) or AT_H35L_ (*in vitro*) groups.

### Statistical Analyses

Data were analyzed using *t* tests, Mann-Whitney tests, analysis of variance (ANOVA) followed by Dunnett’s test, or Kruskal-Wallis followed by Dunn’s test. All statistical analyses were performed using GraphPad Prism version 6.0. Histopathological analyses were performed by a pathologist who was blinded to group allocation. A *P* value of ≤0.05 was considered statistically significant.

## Results

### Macrophages are required for clearance of neutrophils

**Figure 1:**
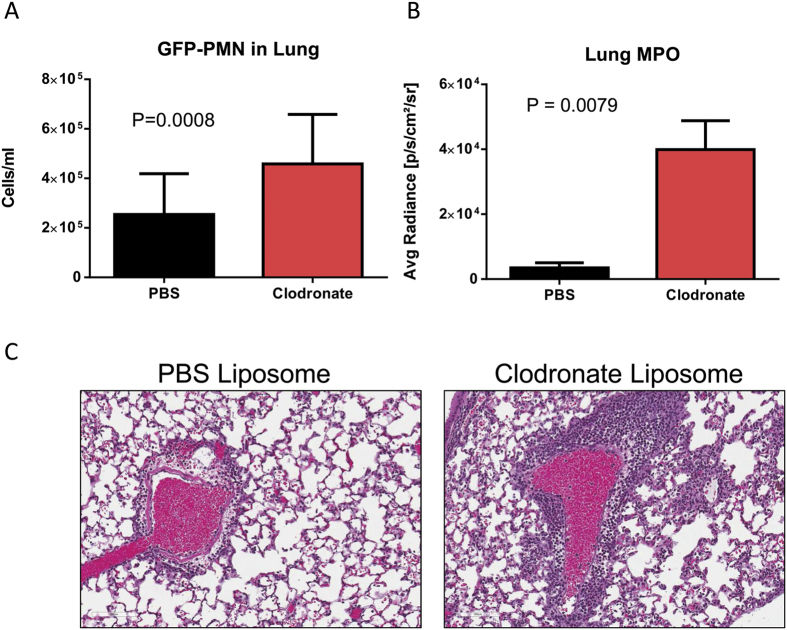
Alveolar macrophage depletion increases neutrophil numbers in the lung 24 h post infections. (**A**) Fluorescent-activated cell sorting (FACS) analysis of the number of GFP labeled neutrophils from BAL of alveolar depleted (Clodronate) or control (PBS) liposome treated mice 24 h intranasal inoculation with GFP-labeled neutrophils. (**B**) MPO activity in lungs of AM-depleted (Clodronate) or control (PBS) treated mice 24 h post *S.aureus* (1e7 CFU SF8300) infection. (**C**) Hemotoxylin and Eosin staining of lungs from AM-depleted (Clodronate) or control (PBS) treated mice 24 h post infection with *S.aureus* (1e7 CFU SF8300). Significance was determined by Mann-Whitney test; data representative of 3 independent experiments (n ≥ 5 mice per group).

Macrophages have been implicated in the clearance of neutrophils from infected lungs^19^. To confirm these previous observations we intranasally inoculated mice with liposomal clodronate to deplete AMs or with PBS liposome control, 24 h prior to intranasal delivery of GFP labeled neutrophils. As previously reported, clodronate liposomes reduced AM numbers by approximately 90% without affecting numbers of recruited inflammatory monocytes or neutrophils (Supplemental Fig. 2)^4^,^17^,^20^. The number of GFP positive neutrophils recovered from the lung 24 h after neutrophil inoculation was measured by flow cytometry. Significantly more neutrophils were recovered from clodronate treated animals as compared to the PBS liposome controls, confirming a role for resident AMs in the removal of neutrophils (Fig. 1A). Control or clodronate liposome treated mice were infected with a sub-lethal inocula (1e7 CFU) of *S. aureus*. Twenty-four hours post infection myeloperoxidase (MPO) and neutrophil elastase (NE) levels were found to be significantly higher and increased numbers of neutrophils were observed by immuno-histochemistry in the lungs of clodronate treated mice as compared to those receiving control liposomes (Fig. 1B,C, Supplemental Fig. 3A–C)). These data suggest that during infection resident AMs contribute to clearance of neutrophils from the lung.

### AT prevents macrophage clearance of neutrophils from the lung

**Figure 2:**
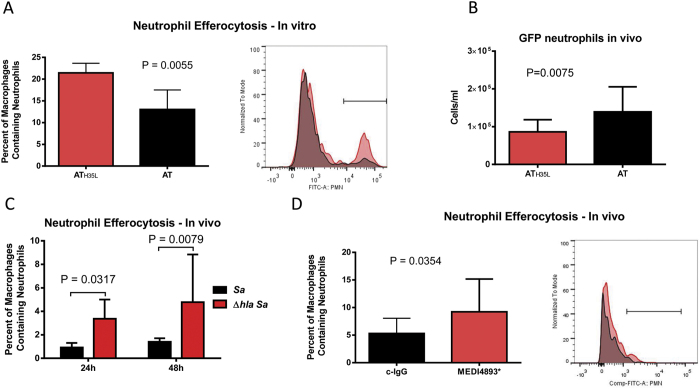
Alpha Toxin (AT) reduces the effercytosis of neutrophils. (**A**) *In vitro* uptake of GFP neutrophils by primary murine AMs in the presence of AT_H35L_ or AT (0.1 μg/mL) measured by flow cytometry, bar indicates neutrophil positive macrophages. (**B**) Numbers of GFP neutrophils recovered from the BAL of mice 24 h following intranasal instillation of GFP neutrophils along with either AT or AT_H35L_ (0.05 μg). (**C**) FACS analysis of neutrophil efferocytosis by macrophages *in vivo* 24 h and 48 h following infection with either WT *S. aureus* or Δ*hla S.* aureus. (**D**) *In vivo* efferocytosis of GFP neutrophils by macrophages measured by flow cytometry analysis of lung homogenates, bar indicates neutrophil positive macrophages. Mice immunized with either control IgG (c-IgG) or MEDI4893* (15 mg/kg) were infected 24 h later with *S. aureus* (5e7 CFU). Lungs were harvested 24 h post-infection. Significance was determined by Mann-Whitney test. Data representative of 3 independent experiment (**A,C,D** n = 5 mice per group, **B** n = 10 mice per group).

*S. aureus* AT significantly impacts macrophage function, activating inflammasome signaling and impairing bacterial killing^2^,^3^,^4^,^17^. To determine if AT alters macrophage clearance of apoptotic neutrophils, we induced apoptosis in GFP-labeled neutrophils with staurosporin, and subsequently incubated them with alveolar macrophages at a 20:1 ratio (neutrophil:macrophage) in the presence of AT or the inactive mutant toxoid AT_H35L_. Efferocytosis was measured by flow cytometry following a 2 h incubation. The addition of AT, at a concentration previously found to be sublytic for either macrophages or neutrophils, significantly reduced the percentage of neutrophil positive AMs as compared with a control toxoid AT_H35L_ (0.1 μg/ml) (Fig. 2A, Supplemental Videos 1, 2)^17^. Therefore, AT impaired neutrophil efferocytosis by AMs through a process independent of macrophage lysis. Similarly, when neutrophils were intranasally delivered to naïve mice along with AT (0.05 μg) significantly more GFP neutrophils were recovered 24 h later as compared to mice given AT_H35L_ (Fig. 2B). This level of AT did not result in loss of AMs suggesting an alteration in AM function rather than AM killing (Supplemental Fig. 4).

To demonstrate that alveolar macrophages were ingesting neutrophils during infection, GFP neutrophils were injected intravenously into mice 3 h prior to intranasal infection with *S. aureus* or Δ*hla S. aureus.* Twenty-four hours following infection the lungs were removed, the vasculature rinsed with PBS to remove unattached cells (Supplemental Fig. 5), and the percentage of macrophages containing GFP neutrophils was calculated by flow cytometry. The percentage of macrophages containing internalized GFP- neutrophils was significantly higher in mice infected (24 h or 48 h) with Δ*hla S. aureus* as compared with WT *S. aureus* (Fig. 2C). We confirmed an inhibitory role of AT in neutrophil efferocytosis during *S. aureus* infection by injecting mice with AT-neutralizing antibody (MEDI4893*) 24 h prior to infection with WT *S. aureus*, and neutrophil engulfment was again measured by flow cytometry. The percentage of macrophage phagocytosed neutrophils was higher in mice pre-treated with AT neutralizing antibody confirming a role of AT in blocking neutrophil clearance (Fig. 2D).

### AT alters DD1α distribution on the macrophage surface

**Figure 3:**
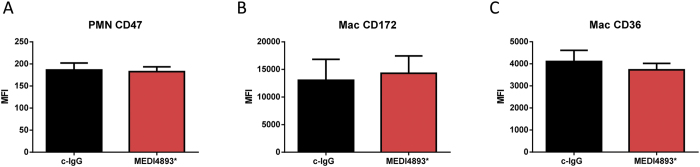
AT neutralization does not impact surface expression of efferocytosis-associated integrins or their receptors. Mice immunized with either control IgG (c-IgG) or MEDI4893* (15 mg/kg) were infected 24 h later with *S. aureus* (5e7 CFU). Lungs were harvested 24 h post-infection. FACS analysis of (**A**) receptor CD47 on lung neutrophils, (**B**) receptor CD172 on alveolar macrophages, and (**C**) CD36 on alveolar macrophages. Data are representative of at least 3 independent experiments (n = 10 mice per group).

Phagocytosis of *S. aureus* by neutrophils is thought to alter expression of surface proteins such as CD47, and consequently reduce neutrophil efferocytosis^15^. We therefore analyzed expression of surface receptors on neutrophils (CD11c^−^CD11b^+^ Ly6G^+^) and AMs (CD11c^+^ CD11b^−^SiglecF^+^) in the lungs of mice passively immunized with MEDI4893* or control antibody and infected for 24 h with *S. aureus*. Neutralization of AT with MEDI4893* did not change neutrophil expression of CD47, nor did it alter AM expression of CD36 or CD172 (Fig. 3A–C), suggesting that expression of surface markers associated with promoting or inhibiting efferocytosis were not altered by AT.

**Figure 4:**
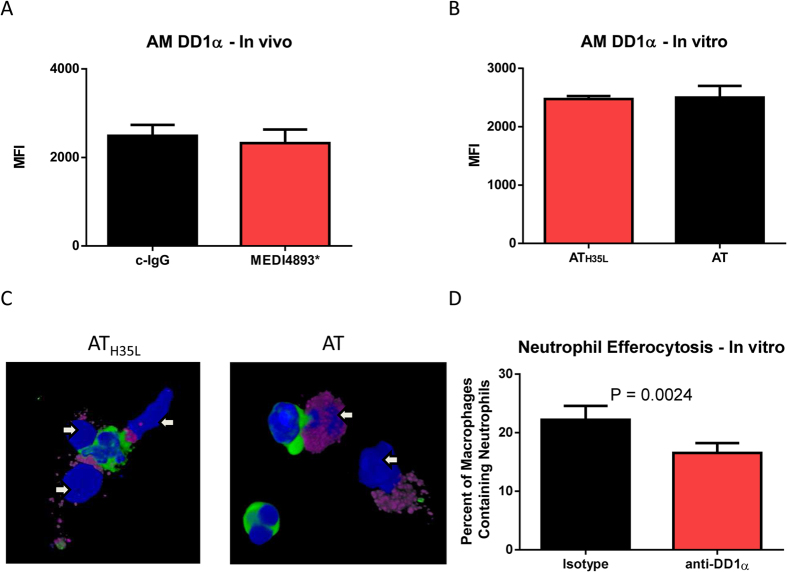
Alpha toxin alters DD1α membrane distribution. (**A**) FACS analysis of neutrophil uptake by AM in the presence of c-IgG or neutralizing antibody against DD1α. (**B**) FACS analysis of DD1α expressed by AM recovered from BAL of mice immunized (24 h prior to infection) with c-IgG or MEDI4893* and then infected with *S. aureus* (5e7 CFU). BAL was harvested 24 h post-infection. (**C**) Confocal images of the DD1α (purple) synapse formed between AM (arrow) and neutrophils (green) in the presence of 0.1 ug/ml of AT or AT_H35L_. Significance was determined by Mann-Whitney test. (**D**) FACS analysis of DD1α expressed by purified AM in the presence of 0.1 ug/ml of AT or AT_H35L_. Data representative of at least 3 independent experiments (**A**,**C)** n = 5 replicates per group, (**B**) n = 10 mice per group, (**D**) 2 biologic replicates, 3 images per replicate collected per experiment).

Analysis of anti-tumor macrophages has recently identified C10orf54 (DD1α or PD-1H), a p53 regulated surface receptor expressed by both macrophages and neutrophils, as a novel protein involved in efferocytosis^21^. We analyzed the effect of AT on DD1α surface expression on macrophages and neutrophils *in vitro* and *in vivo*. AT did not influence the degree to which DD1α was expressed on the surface of macrophages (Fig. 4A,B), however confocal microscopy of *in vitro* macrophage neutrophil interaction revealed that AT altered the surface distribution of DD1α, redirecting it from the site of macrophage-neutrophil interaction to a more disorganized, diffuse surface expression around the cell (Fig. 4C). In our *in vitro* system, we also found that neutralizing DD1α reduced neutrophil efferocytosis to a similar degree as was observed in AT treated cells (Figs 2A and 4D). We postulate that redistribution of DD1α influences the efferocytosis process without a significant change in overall surface expression.

### AT reduces expression of CCN1

**Figure 5:**
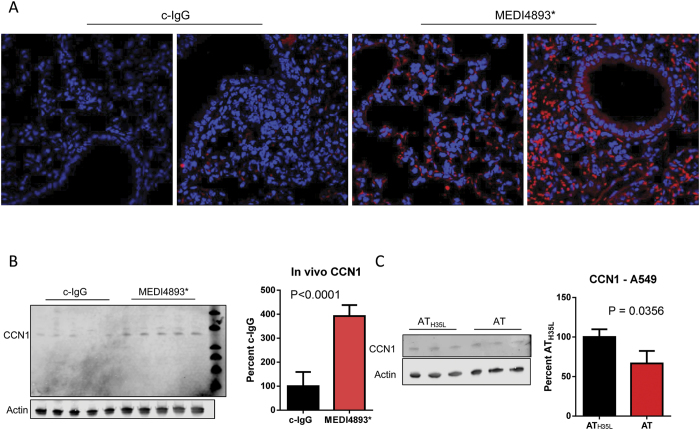
Alpha toxin (AT) reduces CCN1 expression. (**A**) Immunofluorescent staining of CCN1 (red) and nuclei (blue) in lung sections from mice immunized (−24 h) with MEDI4893* or c-IgG and then infected with *S. aureus* for 24 h. (**B**) Western blot analysis of CCN1 expression in lung homogenates from mice immunized (−24 h) with MEDI4893* or c-IgG and then infected with *S. aureus* for 24 h. (**C**) Western blot analysis of CCN1 expressed in A549 epithelial cells incubated with AT or AT_H35L_ (1 μg/ml, 2 h). Significance was determined by Mann-Whitney test. Data representative of at least 3 independent experiments (**A,B** n = 5 mice per group, **C** n = 3 replicates per group).

Cystine rich angiogenic inducer 61 (CYR61 or CCN1) has been shown to promote neutrophil clearance and wound healing in the skin^22^. We analyzed its expression in lungs of mice passively immunized with MEDI4893* and infected with *S. aureus* for 24 h. Immunofluorescent staining of lung slices demonstrated increased CCN1 protein in the lungs of mice treated with MEDI4893* as compared with c-IgG treated mice (Fig. 5A, Supplemental Fig. 6). This observation was confirmed by western blot analysis of lung homogenates (Fig. 5B). Alveolar epithelial cells in the lung are major producers of CCN1, and the immuno-staining of infected lung slices suggest that much of the CCN1 in the lung is present on alveolar epithelial surfaces^23^,^24^,^25^. We utilized a human epithelial cell line, A549 cells, to test *in vitro* if AT would reduce levels of CCN1 in epithelial cells. A549 cells were treated with a sublytic dose AT dose (0.1 μg/ml) or AT_H35L_ for 2 h and CCN1 protein levels were measured by western blot. Significantly less CCN1 was recovered from cells treated with AT as compared to the control toxoid (Fig. 5C). These data confirm our *in vivo* observation that AT reduces the amount of CCN1 in the epithelium.

## Discussion

Inflammation plays a critical physiological role during infection or injury, initiating leukocyte recruitment and coordinating activation of antimicrobial signaling pathways. Although meant to curtail infection, inflammatory dysregulation resulting in excessive inflammation can lead to tissue damage and eventually organ failure. In these studies, we demonstrated that *S. aureus* alpha toxin (AT) impairs the ability of alveolar macrophages to clear neutrophils from the airway, and have provided two potential mechanisms through which AT can interrupt this pathway.

Neutrophils have long been appreciated as an essential component of the innate immune response to respiratory pathogens, however when left unchecked neutrophils can contribute to local tissue damage. Clinically, inflammatory respiratory diseases such as acute respiratory distress syndrome (ARDS), chronic obstructive pulmonary disease (COPD), and cystic fibrosis are all associated with increased numbers of neutrophils in the airway^26^,^27^,^28^. Neutrophils are an especially short lived immune cell and rapidly activate apoptotic or necrotic death pathways following ingestion of bacteria. Apoptosis is a non-inflammatory mode of cell death characterized by nuclear and cytoplasmic condensation and cellular fragmentation into membrane bound fragments^29^. Necrosis, however, involves rapid plasma membrane permeablization and the release of cellular contents (DAMPS) provoking a strong inflammatory response that can contribute to tissue damage and mortality^30^. *S. aureus* is thought to promote necrotic death, and *Rip3*^−/−^ mice, in which necroptosis (programmed necrosis) pathways are inhibited, are protected from *S. aureus* pneumonia^4^,^15^. Interestingly, depletion of alveolar macrophages did not impair bacterial clearance in *Rip3*^−/−^ mice, as it did in WT controls, suggesting that RIP3 dependent death of other cells, such as neutrophils, contributes to prolonged infection.

Optimal clearance of neutrophils is reliant on macrophage CD36 expression, as evidenced by CD36 dependent removal of neutrophils from the skin of mice during *S. aureus* dermonecrosis^31^. While CD36 is necessary for dermal healing and contributes to phagocytosis of *S. aureus*, we did not observe AT dependent alterations in CD36 expression on macrophages in the lung^32^. Similarly, AT did not alter expression of CD47 or CD172 (the “don’t eat-me signaling axis”), suggesting that other mechanisms underlie AT’s effect on neutrophil clearance. Recent work has described a role for CCN1 and DD1α in clearance of dying cells by macrophages, and we found significant effects of AT on each^21^,^22^. CCN1 is expressed and secreted by epithelial cells and in the extracellular space it binds to phosphatidylserine on the surface of apoptotic cells and integrins (α_V_β_3_/α_V_β_5_) on macrophages. CCN1 subsequently activates Rho signaling in the macrophage to initiate uptake. Conversely, DD1α driven efferocytosis is independent of phosphatidylserine. DD1α is expressed by both the dying cell and macrophages, and uptake of the dying cell is initiated upon DD1α-DD1α cross binding. We found that full length CCN1 protein expression in the lung was reduced and cleaved CCN1 protein levels increased in the presence of AT. Meanwhile DD1α bridge formation between neutrophils and macrophages was altered by AT, which was sufficient to alter neutrophil efferocytosis *in vitro*. While the primary mechanism through which AT influences the efferocytosis pathway has yet to be identified, our data suggest that AT is capable of acting through multiple pathways to impair neutrophil clearance.

CCN1 and DD1α, have activity beyond their role in the efferocytosis process. CCN1 has multiple signaling domains, which mediate cell adhesion and spreading, promote angiogenesis and wound repair, as well as activate inflammatory signaling pathways and influence classical macrophage activation^33^,^34^,^35^. Furthermore, CCN1 exerts an anti-inflammatory response during bacterial infection reducing MIP-2, TNF-α, and neutrophil infiltration, without altering the infiltration of macrophages or dendritic cells^24^,^36^. Deletion of *Cyr61* which encodes CCN1 is an embryonic lethal mutation^37^. DD1α is a potent inhibitor of T-cell activation^21^. *S. aureus* induces a robust inflammatory T-cell response through expression of numerous super-antigens, and mice lacking T-cells were more proficient at clearing *S. aureus* from their lungs^38^. While beyond the scope of the current study, it is possible that alterations in the functional capacity of these two proteins has a significant effect on essential immune signaling pathways, further blunting the ability of the host to clear airway bacteria.

The effect of AT on the efferocytosis of neutrophils was inhibited when animals were immunized with anti-AT mAb MEDI4893*. This mAb is currently in clinical trials for the prevention of *S. aureus* pneumonia. While the mechanism of protection was thought to be inhibition of cell lysis by AT, data form the current study, and those previously published by our group, suggest that AT influences a variety of immune functions^17^. We have previously reported that AT alters maturation of endosomes, inhibiting the capacity of macrophages to kill phagocytosed bacteria^17^. The current communication builds upon this work, and demonstrates that macrophage regulatory functions are also impaired by *S. aureus* AT in a mouse pneumonia model. Therefore, neutralization of this single virulence factor can positively influence many facets of the immune response during *S. aureus* infection.

In conclusion, we demonstrate that *S. aureus* AT reduces clearance of neutrophils by AMs in infected lungs, and have identified two pathways through which AT influences efferocytosis of neutrophils. We propose that impairing clearance of these cells increases lung damage, and potentially increases the capacity of *S. aureus* to colonize the lung. Neutralizing the effects of AT with a monoclonal antibody, such as MEDI4893*, reversed this function of AT and promoted clearance of both *S. aureus* and neutrophils from the airway, providing a new mechanism by which neutralization of AT ameliorates *S. aureus* necrotizing pneumonia.

## Additional Information

**How to cite this article**: Cohen, T. S. *et al*. *S. aureus* blocks efferocytosis of neutrophils by macrophages through the activity of its virulence factor alpha toxin. *Sci. Rep.* **6**, 35466; doi: 10.1038/srep35466 (2016).

## Supplementary Material

Supplementary Information

Supplementary Video 1

Supplementary Video 2
