# Supplementary material for: S. aureus blocks efferocytosis of neutrophils by macrophages through the activity of its virulence factor alpha toxin
Source: Sci Rep. 2016 Oct 14;6:35466. doi: 10.1038/srep35466 (PMC5064327; doi:10.1038/srep35466)
Supplement: Supplementary Information [file srep35466-s1.pdf]

Supplemental Information

S. aureus blocks efferocytosis of neutrophils by macrophages through the activity of its virulence factor alpha toxin

Taylor S. Cohen[1], Omari Jones-Nelson[1], Meghan Hotz[1], Lily Cheng[2], Lloyd S. Miller[3], JoAnn Suzich[1], C. Kendall Stover[1], Bret R. Sellman[1*]

Department of Infectious Disease, Medimmune, LLC[1] One MedImmune Way, Gaithersburg, MD 20878

Department of Translational Science, Medimmune, LLC[2] One MedImmune Way, Gaithersburg, MD 20878

Department of Dermatology, John Hopkins University School of Medicine, Baltimore MD 21231

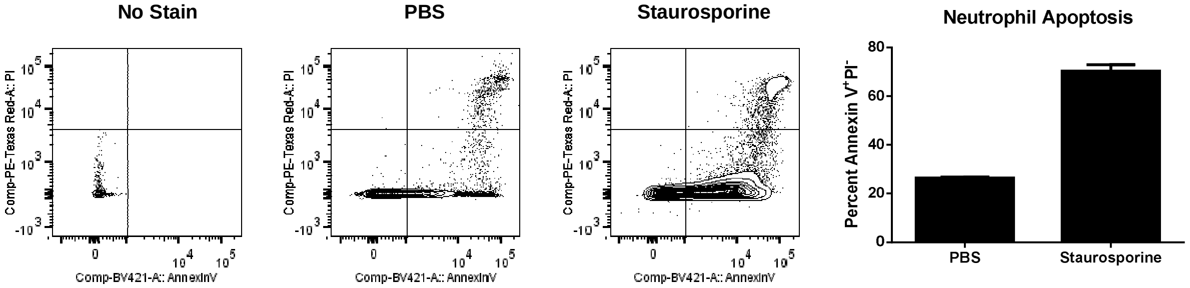

Supplemental Fig. 1. Staurosporine induced neutrophil apoptosis. Flow cytometry analysis of neutrophils following 4h incubation with staurosporine or PBS control. Apoptosis was measured as the percent of cells Annexin V^+ Propidium iodide $(PI)^-$. Representative of 2 independent experiments, n=4 per group.

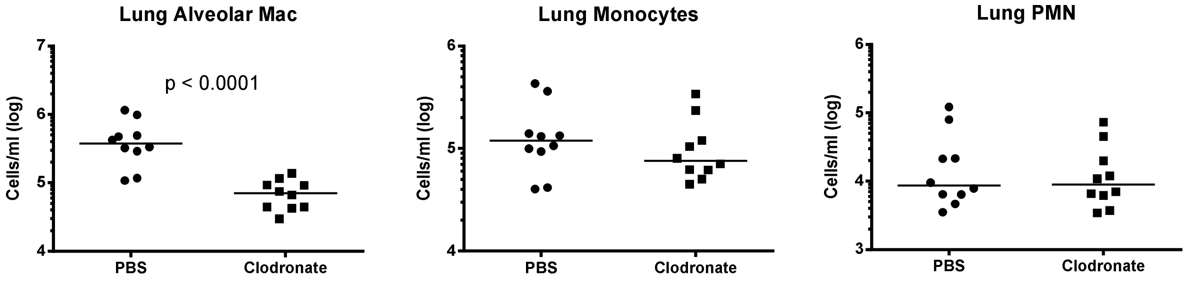

Supplemental Fig. 2. Alveolar macrophage depletion by liposomal clodronate. Numbers of alveolar macrophages ($CD11c^{+}CD11b^{-}SiglecF^{+}$), inflammatory monocytes ($CD11c^{-}$ $CD11b^{+}Ly6C^{+}$ $Ly6G^{-}$) and neutrophils ($CD11c^{-}CD11b^{+}Ly6C^{+}$ $Ly6G^{+}$) in the lungs of mice 24h following intranasal PBS or clodronate liposomes determined by flow cytometry. Significance was determined by Mann-Whitney test; representative of greater than 3 independent experiments.

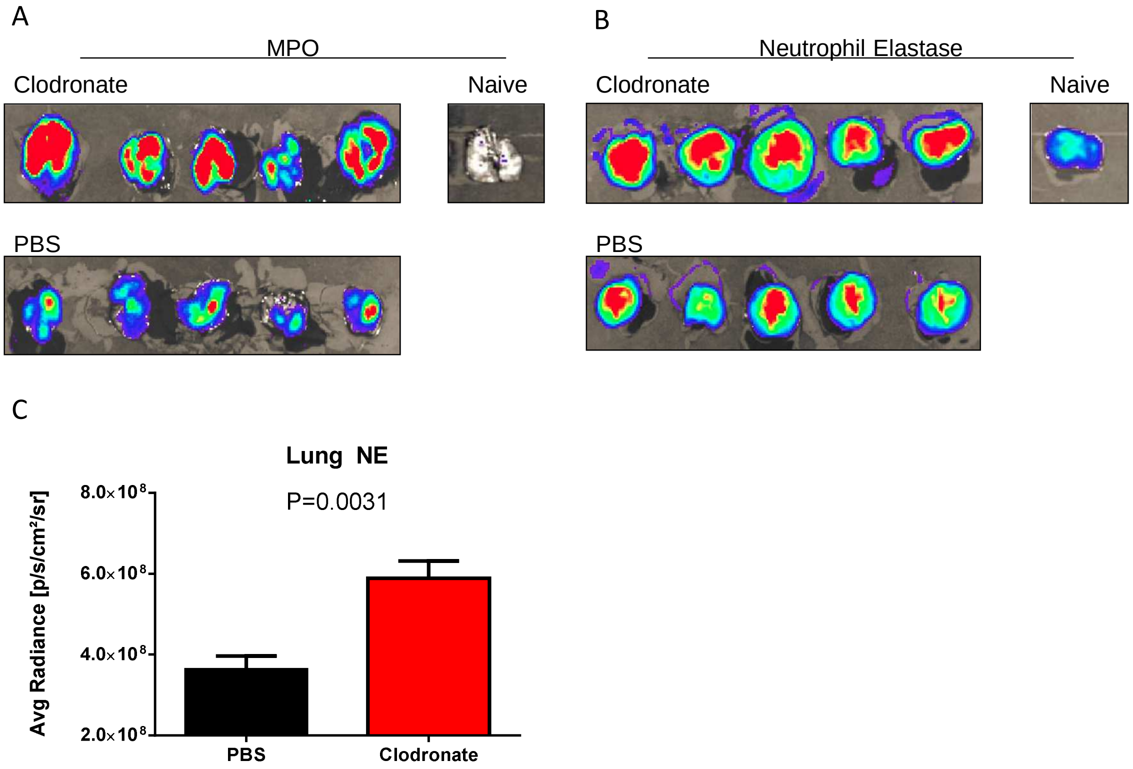

Supplemental Fig. 3. Increased MPO and NE activity in *S. aureus* infected mouse lungs lacking alveolar macrophages. (A) MPO chemiluminescent probe activity of infected lungs harvested 24h post *S. aureus* (1e7 CFU) infection from AM-depleted (Clodronate) or control (PBS) liposome treated mice. **(B)** NE fluorescent probe activity of infected lungs harvested 24h post *S. aureus* (1e7 CFU) infection from AM-depleted (Clodronate) or control (PBS) liposome treated mice. **(C)** NE activity in infected lungs harvested from clodronate or control liposome treated mice 24h post *S. aureus* (1e7 CFU) infection. Significance was determined by Mann-Whitney test. Data representative of 3 independent experiments (n = 4 mice per group).

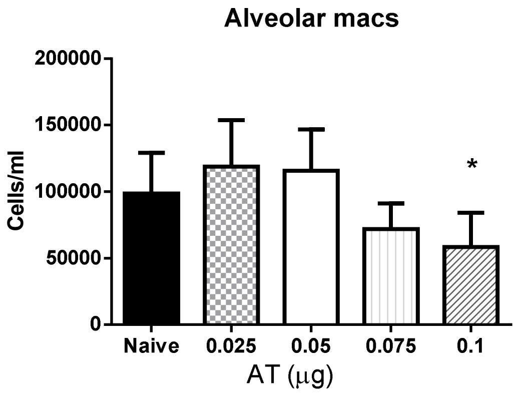

Supplemental Fig. 4. Effect of AT on alveolar macrophage number and efferocytosis
Number of alveolar macrophages in lung homogenates 24h following nasal instillation of AT at various concentrations. Significance was determined by Mann-Whitney test, * indicates significantly different than naive. Data are representative of at least 3 independent experiments (n = 5 mice per group).

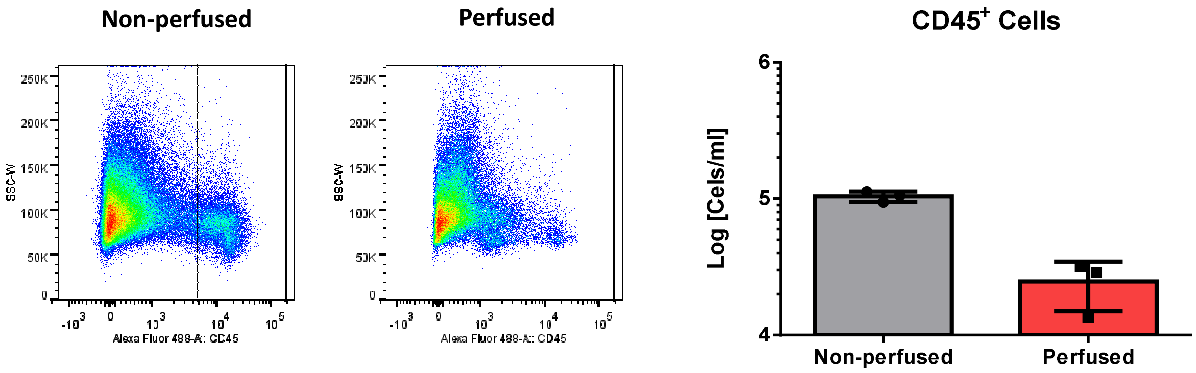

Supplemental Fig. 5. Vascular perfusion removes CD45$^+$ cells. Naïve mice were injected (IV, 100 µL) with 10 µg FITC anti-CD45 antibody and sacrificed 5 min later. The lungs were then isolated with or without vascular perfusion with PBS, homogenized through a 40µm filter and the number of stained CD45$^+$ cells were counted by flow cytometry.

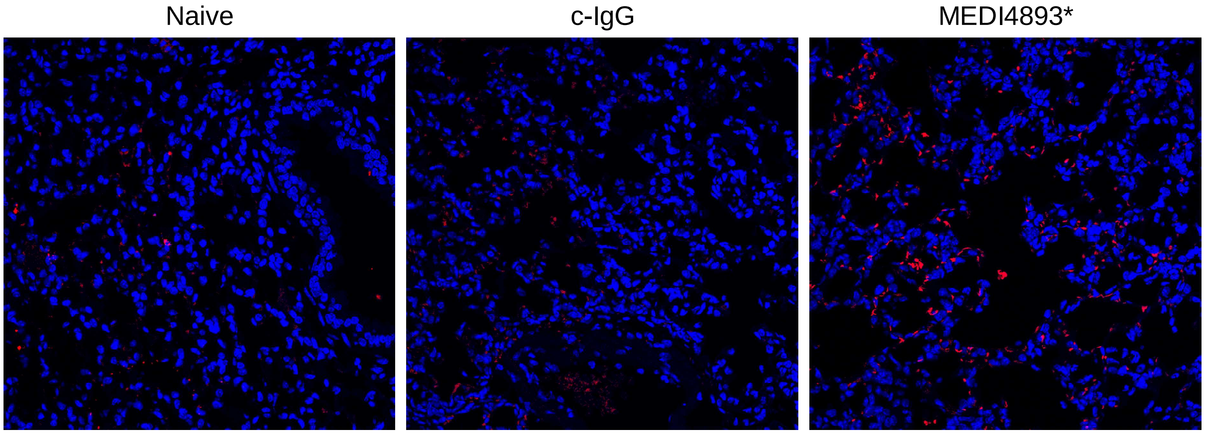

Supplemental Fig. 6. CCN1 expression in the lung. (A) Immunofluorescent staining of CCN1 (red) and nuclei (blue) in lung sections from naïve mice and mice immunized (-24h) with MEDI4893* or c-IgG and then infected with *S. aureus* for 24h.

Supplemental Video 1. Macrophage (grey) efferocytosis of a neutrophil (green) in presence of AT_{H35L} (0.1 mg/mL).

Supplemental Video 2. Macrophage (grey) efferocytosis of a neutrophil (green) in presence of AT (0.1 mg/mL).
